# Correlation Between Individual Child-Level Antibiotic Consumption and Antibiotic-Resistant Among Commensal *Escherichia coli*: Results from a Cohort of Children Aged 1–3 Years in Rural Ujjain India

**DOI:** 10.2147/IDR.S372093

**Published:** 2022-10-28

**Authors:** Shweta Khare, Vishal Diwan, Ashish Pathak, Manju Raj Purohit, Cecilia Stålsby Lundborg

**Affiliations:** 1 Health Systems and Policy (HSP): Medicines, Focusing Antibiotics, Department of Global Public Health, Karolinska Institutet, Stockholm, 171 77, Sweden; 2 Department of Public Health and Environment, Ruxmaniben Deepchand Gardi Medical College, Ujjain, Madhya Pradesh, 456006, India; 3 Division of Environmental Monitoring and Exposure Assessment (Water and Soil), ICMR—National Institute for Research in Environmental Health, Bhopal, Madhya Pradesh, 462030, India; 4 Department of Pediatrics, Ruxmaniben Deepchand Gardi Medical College, Ujjain, Madhya Pradesh, 456006, India; 5 Department of Pathology, Ruxmaniben Deepchand Gardi Medical College, Ujjain, Madhya Pradesh, 456006, India

**Keywords:** Escherichia coli, Kirby Bauer disk diffusion method, healthcare-seeking behaviour, common childhood illness, caregivers, under-5 children, antibiotic prescribing, antibiotic resistance, rural population, India

## Abstract

**Background:**

The global expansion of antibiotic-resistant bacteria is a serious concern and is increasing worldwide in both pathogenic and commensal bacteria. The study determined the correlation between individual child-level antibiotic consumption and antibiotic resistance among the commensal *Escherichia coli (E.coli)* in a cohort of 125 children in rural Ujjain, India.

**Methods:**

During a two-year period between August 2014 and September 2016, stool samples were collected at seven-time points from a cohort of 125 children; aged 1–3. A total of six colonies of *E.coli* per stool sample were collected for antibiotic susceptibility testing. Antibiotic consumption data was collected during the healthcare-seeking follow–up done during the same period. At each of the seven-time points correlation between antibiotic consumption (Defined Daily Dose-DDD/100 patient-days) and antibiotic resistance (number of resistant isolates) was analyzed independently using the Spearman correlation coefficient. Further, mixed-effects logistic regression models were built to study correlation between child-level consumption of penicillin with the number of *E.coli* isolates resistant to ampicillin, consumption of cephalosporin with resistance to cefotaxime and ceftazidime, consumption of fluoroquinolones with resistance to nalidixic acid and consumption of cotrimoxazole with resistance to cotrimoxazole.

**Results:**

Out of 756 illness episodes reported in 125 children 42% were with antibiotic prescriptions and reported a total antibiotic consumption of 55DDD/100 patient-days. The most common antibiotics used were cefixime (J01DD08;72 DDD/100patient/days) followed by ofloxacin (J01MA01;51DDD/100patient-days), cefpodoxime (J01DD13;38DDD/100patient-days) and amoxicillin (J01CA04;28DDD/100patient-days). The highest percentage of resistance was found to the ampicillin (67%) followed by nalidixic acid (52%) and cefotaxime (44%) and when summarized, more than 90% were resistant to cefotaxime, ceftazidime, and co-trimoxazole in commensal *E.coli* isolates. The consumption of cephalosporins showed weak positive correlation with the resistance to cefotaxime (Coefficient±SE=0.13 ± 0.09,*p*<0.001).

**Conclusion:**

Our findings showed no correlation between individual-level antibiotic consumption and resistance development in commensal *E.coli* in a rural community environment.

## Introduction

Antibiotic resistance is a major public health problem worldwide since the number of bacteria that are resistant to multiple drugs is increasing at an alarming rate.^1^ Bacterial resistance strains are not just prevalent in hospital settings; rather, a wide diversity of resistant strains can be found in the community.^1^ Each year, it is estimated that over 50,000 babies die of sepsis caused by bacteria resistant to first-line medicines.^1^ While precise population burden estimates are unavailable, it is believed that neonates and the elderly will be most impacted. By the year 2050, it is estimated that antimicrobial resistance will claim two million lives in India.^2^

Antibiotic resistance is caused by a multitude of factors, including current medical practices of health-care providers, antibiotic usage patterns, community perceptions, over-the-counter sales of antibiotics, socio-demographic characteristics and hygiene and clean water practices.^3–5^ One of the most significant reasons contributing to the rise of antibiotic resistance is the misuse and overuse of antibiotics in the community. This can be estimated by regular monitoring, which is lacking in community settings, especially in rural areas, which are home to around 70% of the total population of India.^6^ Another reason is the high burden of infectious diseases. Infectious diseases continue to be the primary cause of death in children under 5 in India. Acute infections like bacterial sepsis, respiratory tract infections, and diarrhoea are the top causes of death in children under the age of five.^6^ A high infectious disease burden is reported as the biggest threat and it showed inappropriate and irrational use of antibiotics, which led to increased antibiotic resistance.^7^ An individual’s healthcare-seeking behaviour (HSB) is also influenced by all of these elements. When it comes to healthcare-seeking in India, many people especially in rural areas start with home care, and/or the informal health-care system, which frequently involves the inappropriate use of medication, especially antibiotics, a major contributor to antibiotic resistance.^8–11^ Physiological and sociocultural factors, such as community exposure and ambulatory care, contribute significantly to an increase in antibiotic resistance.^12^,^13^ Public data from Indian communities are scarce, even though there is a widely documented high-level resistance in India. Antibiotic stewardship policies can be more effective when they are based on a clear understanding of the link between usage and resistance. This study’s results bridge the gap in our understanding of the prevalence of antibiotic resistance and its association with antibiotic consumption in a rural community setting.

*Escherichia coli (E.coli)*, a common gastrointestinal inhabitant, and a key player in the spread of antibiotic resistance have been used to monitor and track the development of antibiotic resistance in study cohort. Faecal flora acts as a reservoir for resistant gene variants.^1^ When commensal gut bacteria like *E.coli* are exposed to antibiotics, they acquire antibiotic-resistant genes, and this resistance may be passed on to a more pathogenic organism.^14^ Several bacteria, including *E.coli*, have been recognized by the World Health Organization (WHO) as the most serious threat to the global spread of antibiotic resistance.^15^ Antibiotic resistance has previously been reported in commensal *E.coli* strains isolated from children who were not sick, animals, and water supplies.^16^ Therefore, this study aims to determine the correlation between individual child-level antibiotic consumption and antibiotic resistance among the commensal *E.coli* in a cohort of 125 children at 7 time-points over the period of 2 years in rural areas of Ujjain, India.

## Materials and Methods

### Study Design

Prospective correlation study, where a prospective analysis of the correlation between individual child-level antibiotic consumption and antibiotic resistance of the commensal *E.coli* cultured from stool samples collected from the cohort of children 1–3 years of age at 7-time points over a period of 2 years from August 2014 to September 2016 was conducted.

### Study Setting

The study was carried out in 6 villages in the Ujjain district of Madhya Pradesh (MP) in central India.^17^ MP is the fifth most populous state in India, with a population of 72.6 million people; with the majority of the population living in rural areas (75%).^18^ Ujjain district has 131 villages. The study villages were selected purposively from within the rural demographic surveillance site (DSS) Palwa of Ruxmaniben Deepchand Gardi Medical College (RDGMC). One of the villages within the DSS was selected and named “Central village” as it had the highest concentration (80%) of health-care providers (both formal and informal). Then, for the selection of the study villages, first, a 5 km zone diameter was created around the central villages and then all the villages within the 5 km zone diameter were enlisted.^17^

### Study Sample

The details of sampling are described in detail elsewhere.^17^ In short, the study included 6 villages. All the samples from the selected villages were analyzed at RDGMC’s central research laboratory (CRL). Villages located within a 5-kilometre distance (aerial distance) of the central village (described above); had a population of at least 500 people; a minimum of 15 children in the age range of 1–3 years are available in the village and had a transport time of less than 45 minutes to the CRL, RDGMC were the criteria used to select the villages for this study. The children aged 1–3 years from within the 6 study villages were selected using the following inclusion criteria: 1) having lived in the village for at least one year; 2) planned to stay in the village for at least two years, and 3) willing to take part in the study after written informed consent from parents/guardians. Simple random sampling based on random number tables was then used to select the 125 children from within the eligible children list.^17^

### Sample Size Calculation

Estimates of proportions from a single batch of 100 children (but equally applicable to antibiotic prescriptions) have a precision of ± 10%. Using a repeated sampling of 100 children at seven-time points has 80% power to detect a linear trend in any observation in the proportion of 0.1 per time unit (step between sampling, negative or positive). The numbers of children included in the cohort were increased to 110 to accommodate for possible attrition over time. The sample size of 110 children gives a sufficiently high precision in estimates and at least an 80% power in comparisons between data points.^17^

### Data Collection

#### Stool Sample Collection and Transportation

Stool sample was collected from the selected child under the supervision of the first author and the trained research assistants during the study period. One stool sample per child included in the cohort (n = 125) was collected at seven-time points at an interval of four months (covering distinct seasons): time point 1 – monsoon 2014 (late June to September), time point 2 – winter 2015 (October to February), time point 3 – summer 2015 (March to June), time point 4 – monsoon 2015, time point 5 – winter 2016, time point 6 – summer 2016 and time point 7 – monsoon 2016.^19^ The stool sampling kit which included: 1) autoclaved plastic container with a wide opening and a spoon; 2) sterile polythene sheet measuring 15*15 cm in dimension (all enclosed in a pre-sterilized polythene zip-lock bag) was used to collect the stool samples. The kits were distributed to the child’s caregivers one day before the stool sample collection. Parents were trained on how to collect stool samples using the stool sampling kit. The training was repeated at all 7-time points before the stool sample collection day. A cold chain of 4–6°C (for 4–5 hours) was maintained from stool sample collection to the transportation of the samples to the CRL, RDGMC, Ujjain for further processing.^16^,^17^,^20^

#### Preparation of the Samples

Microbiological processing started immediately after receiving the samples in the CRL. The samples were plated on selected and differentiated HiCrome^®^ coliform chromogenic media (HiMedia Laboratories Pvt. Ltd., Mumbai, India) and *E.coli* colonies were identified (blue-violet colonies). Six *E. coli* colonies were isolated, processed, validated using polymerase chain reaction (PCR), purified, and kept for further examinations by inoculating at 37°C for 24 hours on chromogenic agar.^17^ The Kirby-Bauer disc diffusion technique,^21^ using disc strengths recommended by the Clinical and Laboratory Standards Institute (CLSI),^22^ was used for conducting antibiotic susceptibility test (AST) on all six verified *E. coli* isolated from each stool sample. The isolates were tested against the following five antibiotics: ampicillin, cefotaxime, ceftazidime, nalidixic acid and co-trimoxazole (Table 1). Antibiotics were chosen in accordance with the CLSI standards and from the list of common antibiotics used in the hospital attached with RDGMC for gram-negative coliform infections. Two technical specialists independently assessed the diameter of the inhibitory zones to the closest millimeter. The process was repeated if there was a difference of more than three millimeters between two readings. If there was still a disparity in the zone diameter, the CLSI recommended minimum inhibitory concentration approach was used to confirm the susceptibility pattern. All isolates with readings of intermediate susceptibility were regarded as resistant for calculations.^17^

**Table 1:** Antibiotics and Their Respective Class for Which the Antibiotic Susceptibility Test Was Applied

Name	Class
Ampicillin	*Penicillins*
Cefotaxime	3rd generation *cephalosporin*
Ceftazidime	3rd generation *cephalosporin*
Nalidixic acid	Other *fluoroquinolone*
Co-trimoxazole (Trimethoprim/sulfamethoxazole)	*Folatepathway inhibitors*

#### Individual Child-Level Antibiotic Consumption

The data on the individual child-level antibiotic consumption was simultaneously collected during the study period by following the HSB of the mother of cohort of the 125 children.^17^,^23^ The HSBs of the mothers for the acute illnesses in the children were documented using ‘HSB diaries’, with twice-weekly follow-ups (ie, with a recall period of 2 days) of the children for 113 weeks during the study period, done by the first author along with 6 research assistants. The HSB diary documented the route taken by a caregiver in seeking healthcare for a child’s illness through several health-care systems, reasons for choosing a particular healthcare as well as treatment details, with a focus on antibiotics consumed. All consumed drugs, including antibiotics, were verified during the subsequent biweekly visit. That is, the prescribed dose of the medicine consumed by the child was tallied with the left medicine in the wrapper/bottle.^17^,^23^

### Data Management

**Figure 1:**
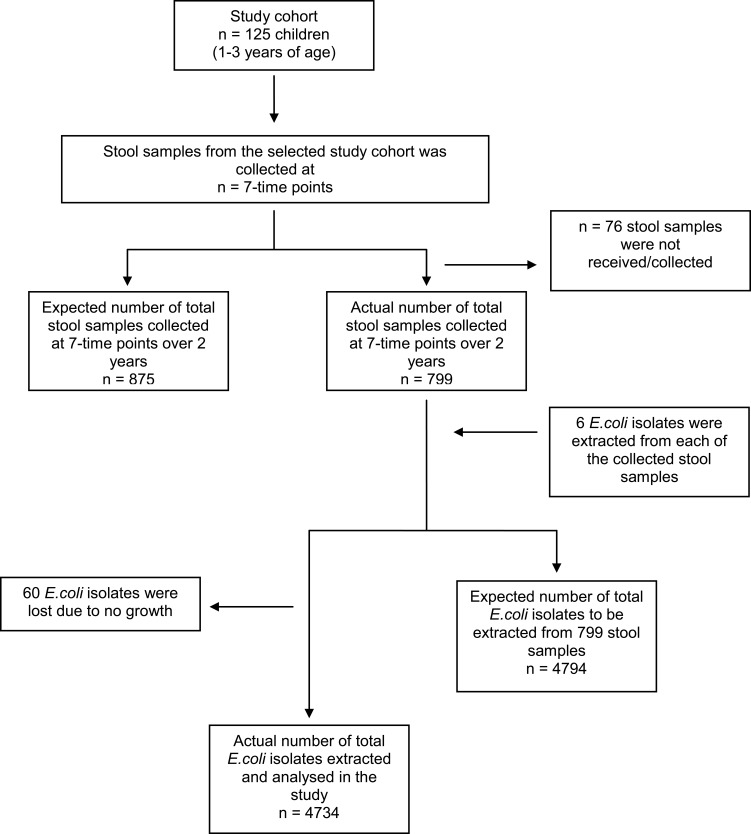
Flow chart describing the study cohort and number of *E.coli* strains isolated from sample and included in the analysis, under study in rural Ujjain, India.

During the study, the household was excluded from the study if they left the study area before all the data could be collected (Figure 1). However, for one particular round of data collection, if the caregiver was unable to provide the child’s stool sample, they were categorized as “Sample Not Obtained” and continued in the cohort. A sample was eliminated from the analysis of a particular time point if it was not received at the CRL or it was not feasible to isolate *E.coli* from the sample (Figure 1).

A drug use (DU) proportion was calculated for each drug as the number of individual antibiotic use divided by the total number of antibiotic uses reported during the study period. The results are presented with antibiotics with more than or equal to 40 DU proportions. The WHO Collaborating Centre for Drug Statistics Methodology, Anatomical Therapeutic Chemical (ATC) classification was used to categorize each given antibiotic with the defined daily dose (DDD) according to the fifth level of the ATC classification, J01 (antibacterial for systemic use).^24^ All DDDs were calculated and adjusted to per 100 patient-days and were also adjusted for the pediatric doses.^25^

The resistance to a single antibiotic was identified as either an intermediate or resistant isolate and recorded as a result of the AST. Isolates with “intermediate” resistance were considered “resistant” for the analysis.

### Statistical Analysis

Data were analyzed using Stata V.14.1 (Stata, College Station, Texas). Frequency and percentages were presented for categorical data. Resistance to selected antibiotic class and antibiotic consumption were analyzed over time points (time-variable) using linear regression individually with resistance and antibiotic consumption as dependent variable and time points as independent variable. Shapiro–Wilk for normality test was used to test the normal distribution of data.

#### Correlation Between Antibiotic Resistance and Consumption

Spearman correlation coefficient was used to look for the association between individual child-level penicillin consumption with ampicillin resistance, cephalosporin consumption with cefotaxime and ceftazidime resistance, fluoroquinolones consumption with nalidixic acid resistance and cotrimoxazole consumption with cotrimoxazole resistance. Further, the association between the above pairs of antibiotics and corresponding resistance was explored using mixed-effects logistic regression models. In total 5 mixed-effects logistic regression models were used to assess the correlation between individual child-level penicillin, cephalosporin, fluoroquinolones and cotrimoxazole consumption (DDD/100 patient-days) as explanatory variable (independent variable) and number of *E.coli* isolates resistant to ampicillin, cefotaxime, ceftazidime, nalidixic acid and cotrimoxazole as the outcome variable (outcome variable), respectively. In the models, the individual child was considered as a random effect considering the hierarchical data structure (isolates share the same child). Coefficient ± standard error (SE) was calculated and a *p-*value of ≤0.05 was considered statistically significant.

### Ethical Considerations

The study has been approved by the Institutional Ethics Committee of RDGMC, Ujjain (approval number DNR-311/2013). Verbal and written informed consent were taken from the parents and/or legal guardians of the participating children after informing them about the purpose of the study and also that it did not include any invasive or otherwise dangerous procedures. It was made clear to the parent or guardian that their participation was voluntary and that all information provided would be kept strictly confidential. And the children who were found to be in need of medical attention were referred to paediatric services in collaboration with the RDGMC. The study complies with the Declaration of Helsinki.

## Results

A total of 125 children (46% girls and 54% boys) were enrolled in the study with half of the children (n = 62) were under the age of one year, while the other half (n = 63) were between the ages of two and three years. A total of 756 illness episodes were reported in 125 children during the study period. The most common illness reported in the study was acute respiratory tract infections (ARTIs; 59%, 444/756), followed by fever (11%, 80/756) and gastrointestinal infections (10%, 74/756).

Total 799 stool samples were collected from 125 children during 7 time-points (Figure 1). A total of 4794 *E.coli* isolates were predicted to be retrieved from the 799 stool samples; however, 60 (1.3%) *E.coli* isolates could not be extracted due to lack of growth. Therefore, 4734 (98.7%) *E.coli* isolates were studied for their AST (Figure 1).

### Antibiotic Consumption and Resistance

**Figure 2:**
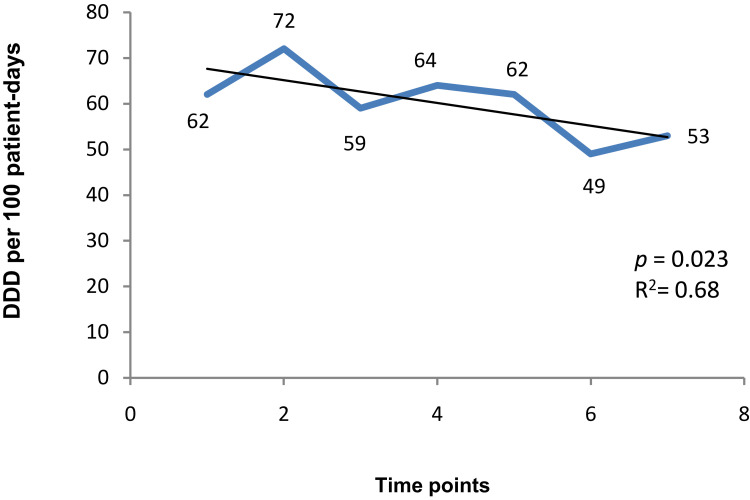
Overall cephalosporin consumption in the study cohort of children of 1–3 years of age at 7-time points, in defined daily doses (DDD) per 100 patient-days, in rural Ujjain, India.

Antibiotics were prescribed in 42% (n = 320) of illness episodes out of a total of 756 illness episodes reported in 125 children. During the study, 337 antibiotic courses were prescribed and consumed. Parenteral (2%, n = 6/337) and topical formulations (6%, n = 20/337) were prescribed less compared to oral formulations (92%, n = 317/337). During the study period, the total antibiotics consumption in the study cohort was 55 DDD/100 patient-days. Overall, cephalosporins (44%, n = 149/337) were the most common class used, followed by penicillins (23%, n = 77/337). The most common antibiotics used were cefixime (J01DD08; 72 DDD/100 patient-days) followed by ofloxacin (J01MA01; 51DDD/100 patient-days), cefpodoxime (J01DD13; 38 DDD/100 patient-days) and amoxicillin (J01CA04; 28 DDD/100 patient-days) (Table 2). During the study period, total antibiotic consumption did not show any significant change over the period of 2 years. A significant decrease in consumption was found for cefixime (*p* = 0.034) and also for the total cephalosporin consumption (*p* = 0.023; Table 2; Figure 2).

**Table 2:** Distribution of Antibiotic Consumption (DDD/100 Patient-Days) for Each of Antibiotic Class at 7-Time Points in Selected Cohort of Children (1–3 Years) During the Study Period

ATC	Antibiotics	Antibiotic Use (DDD/100 Patient-Days) by Time Points							*p* value
		1 Monsoon 2014	2 Winter 2015	3 Summer 2015	4 Monsoon 2015	5 Winter 2016	6 Summer 2016	7 Monsoon 2016	
Total antibiotic consumption		51	60	54	66	51	46	57	0.46
J01C	**Penicillins**	38	43	41	80	32	27	42	0.78
J01CA04	Amoxicillin	28	29	28	50	28	15	29	0.69
J01DD	**Cephalosporins**	62	72	59	64	62	49	53	0.023*
J01DD08	Cefixime	75	100	81	78	68	46	67	0.034*
J01DD13	Cefpodoxime	35	38	34	44	39	100	27	0.53
J01MA	**Fluoroquinolones**	50	43	44	50	55	38	49	0.71
J01MA02	Ciprofloxacin	50	30	25	50	26	25	25	0.2
J01MA01	Ofloxacin	50	50	46	50	60	0	55	0.5
J01EE01	**Co-trimoxazole**	0	113	83	125	125	100	100	0.2

**Notes**: Cephalosporins includes cefixime, cefpodoxime and cefotaxime; penicillinsincludes ampicillin, amoxicillin, amoxicillin/clavulanate and ampicillin/cloxacillin; fluoroquinolones includes ofloxacin, ciprofloxacin, norfloxacin and levofloxacin; *Significant p value <0.05.

The obtained prevalence of antibiotic resistance per isolate to the selected antibiotic class and individual antibiotic by seven-time points are presented in Table 3. The highest rate of resistance was found for ampicillin (67%) followed by nalidixic acid (52%) and cefotaxime (44%). The lowest rates of resistance were found for co-trimoxazole (27%). Every child in the study had at least one isolate resistant to ampicillin and nalidixic acid. Cefotaxime, ceftazidime, and co-trimoxazole have all been shown to have resistance rates greater than 90%. No significant changes in proportions of resistance for the above antibiotics over a 2-year period (time-variable) could be identified.

**Table 3:** Percentage Distribution of *E.coli* Isolates Resistant to the Selected Antibiotic Class at 7-Time Points

Antibiotics	Resistant Isolates (R+I)*n (%)	Resistant Isolates by Time Points							*p* value
		1 N=632 n (%)	2 N=655 n (%)	3 N=675 n (%)	4 N=704 n (%)	5 N=676 n (%)	6 N=699 n (%)	7 N=693 n (%)	
Ampicillin Resistant	3190 (67)	379 (60)	365 (56)	448 (66)	377 (54)	339 (50)	671 (96)	611 (88)	0.12
Cefotaxime Resistant	2062 (44)	313 (50)	253 (39)	322 (48)	318 (45)	263 (39)	260 (37)	333 (48)	0.55
Ceftazidime Resistant	1834 (39)	277 (44)	219 (33)	280 (41)	217 (31)	259 (38)	256 (37)	326 (47)	0.69
Nalidixic acid Resistant	2459 (52)	357 (56)	352 (54)	327 (48)	366 (52)	342 (51)	324 (46)	391 (56)	0.57
Co-trimoxazole Resistant	1292 (27)	163 (26)	134 (20)	205 (30)	205 (29)	176 (26)	169 (24)	240 (35)	0.25

**Notes**: N-total number of *E.coli* isolates tested for resistance.

**Abbreviations**: n, number of resistant isolates; *R+I, Resistant plus intermediate isolates.

### Correlation Between Antibiotic Consumption and Resistance

Spearman correlation coefficient revealed significant association for individual child-level consumption of cephalosporins (60 DDD/100 patient-days) with cefotaxime resistance (2062 resistant isolates) (ρ = 0.04; 95% confidence interval (CI), 0.01–0.07; *p value* = 0.004). However, no statistically significant correlation was identified for penicillin consumption (39 DDD/100 patient-days) with ampicillin resistance (3190 resistant isolates; ρ = −0.08; 95% confidence interval (CI), −0.11 - −0.05; *p value* = 0.07); cephalosporins consumption (60 DDD/100 patient-days) with ceftazidime resistance (1834 resistant isolates; ρ = 0.02; 95% CI, −0.01–0.05;*p value* = 0.232); fluoroquinolones consumption (94 DDD/100 patient-days) with nalidixic acid resistance (2459 resistant isolates; ρ = −0.02; 95% CI, −0.04–0.01; *p value* = 0.23) and co-trimoxazole consumption (108 DDD/100 patient-days) with co-trimoxazole resistance (1292 resistant isolates; ρ = −0.001 [95% CI, −0.04–0.01]; *p value* = 0.92).

Mixed-effects logistic regression models also showed a weak positive correlation between cephalosporins consumption and resistance to cefotaxime (Coefficient ± SE = 0.13 ± 0.09, *p*<0.001; Table 4). In other words, every 0.13 DDD/100 patient-days increase in the cephalosporin consumption, results in a unit increase of the cefotaxime resistance. No significant correlations were found for penicillins, fluoroquinolones and co-trimoxazole consumption and corresponding resistance.

**Table 4:** Mixed-Effects Logistic Regression Analyses of the Relationship Between the Antibiotic Consumption and Resistance Among Selected Antibiotics in the *E.coli* isolates from 125 Children at 7-Time Points Over the Period of 2 Years

Antibiotic Class Consumption	Antibiotic Tested for Resistance	Mixed-Effects Logistic Regression	
		Coefficient ± SE^#^	p value
Penicillins	Ampicillin	−0.75 (± 0.12)	0.16
Cephalosporins	Cefotaxime	0.13 (± 0.09)	<0.001*
Cephalosporins	Ceftazidime	−0.006 (± 0.09)	0.99
Fluoroquinolones	Nalidixic acid	−0.22 (± 0.14)	0.13
Folatepathway inhibitors	Co-trimoxazole	−0.38 (± 0.30)	0.21

**Notes**: *Significant p value <0.05.

**Abbreviation**: ^#^SE, Standard error.

## Discussion

To our knowledge, this is the first rural community-based study, where a possible correlation between individual child-level antibiotic consumption data and antibiotic resistance among the commensal *E.coli* in a cohort of 125 children in Ujjain, India, has been investigated. We found that out of 756 illness episodes reported in 125 children, 42% of episodes were with antibiotic prescriptions and the total antibiotic consumption was of 55 DDD/100 patient-days. The consumption of cephalosporins was positively correlated with the resistance to cefotaxime.

### Antibiotic Consumption

A seasonal variation in overall antibiotic consumption (DDD/100 patient-days) during a 2-year period in the study cohort was seen. A peak in total antibiotic consumption during the winter 2015 (60 DDD/100 patient-days) and monsoon 2015 (66 DDD/100 patient-days) was seen (Table 2). The peak can be attributed to a rise in the number of illness episodes among the study cohort during winter and monsoon seasons as reported during the HSB follow-ups.^23^ Similar peaks in antibiotic prescribing have been observed from a hospital-based outpatient antibiotic use study in Ujjain.^26^

We have not seen any significant change in the total antibiotic consumption during the study period, but a decrease in antibiotic consumption was seen after Monsoon 2015. However, a significant decrease was found only in the consumption of the cephalosporin group of antibiotics. This could be explained by the Hawthorne effect.^27^ We monitored the illness episodes in the cohort and also the prescriptions provided by the health-care providers to the children included in the study area over a 2-year period. The Hawthorne effect might have reduced prescribing by the health-care providers of the children in the cohort, although no direct intervention was done to influence the prescribing.

We reported 42% antibiotic prescribing in our study between 2014 and 2016. This proportion of antibiotic prescribing is lower than that reported in previous studies done in the same geographical area.^26^,^28–30^ This lowered antibiotic prescribing rates could be due to the fact that earlier studies were health-care facility based as compared to our community-based study. Also, the present study measured antibiotic consumption, which is a better measure compared to antibiotic prescribing data. A lower antibiotic consumption rate in community-based study is expected as many illnesses in the community go untreated mainly due to community perception of the severity of illness episode being less serious.^23^ Also, illness episodes perceived as more serious end up presenting to a health-care provider, which in our rural communities are mostly the informal health-care providers.^23^,^28^ Healthcare-seeking from informal health-care providers typically increases the risk of antibiotic prescribing even for a viral illness.^28^ The majority of the illnesses reported were ARTIs. For the ARTI episodes, in 44% of these cases, no antibiotics were given as no treatment was sought.^23^ However, antibiotics were given in 24% of the ARTI cases, which were typically viral, as the caregivers took the child to the health-care providers, of which 89% of the antibiotic prescriptions were from informal health-care providers.^23^

### Antibiotic Resistance

*E.coli* has been proved to be a useful marker bacterium for studying the development and emergence of antibiotic resistance due to its widespread distribution in the human gut and its susceptibility to commonly used antibiotics.^31^ At any given time, there are a number of *E.coli* strains in the human digestive system. However, there are some strains that are permanent residents of the human digestive tract, while others are only present in specific circumstances.^32^ It is, therefore, unlikely that resistance in one *E.coli* isolate will translate to resistance in all other *E.coli* isolates obtained from the same individual over the course of time. In our study, resistance to ampicillin was found in 67% of isolates, while resistance to nalidixic acid was found in 52%. Ampicillin and nalidixic acid-resistant *E.coli* bacteria were found in all children at one or more time points during the study. On the other hand, when taken together, 90% of the children’s commensal *E.coli* isolates were resistant to atleast one of the following antibiotics: cefotaxime, ceftazidime, and co-trimoxazole. A study conducted in 2018 in the rural areas of the state of Sikkim on pre-school and school-going children showed similar results.^33^ However, these occurrence rates are much higher than the rates reported in the previous studies. One of the previous study conducted in 2013 was done in the same study area and reported resistance rate of 72% in *E.coli* isolates from children aged 3 to 14 years.^12^,^34^,^35^ This high antibiotic resistance rate in central India may be attributed to the rapid proliferation of the antibiotic resistance gene pool over the past 9–10 years, especially in commensal *E.coli*.^33^ Concerningly, such a resistance pattern has a direct impact on the child’s health indicators.^36^

Our study results showed a weak positive correlation between the individual child-level cephalosporin consumption and cefotaxime resistance, which are consistent with the findings of the previous studies which showed a significant correlation between the consumption of 3rd generation cephalosporin and 3rd generation cephalosporin resistance rate in *E.coli*.^37^,^38^ In past years, many studies have examined the association between antibiotic use and the development of resistance and have found that the rate and amount of antibiotic use in the population is a key factor of rising antibiotic resistance in bacteria.^30^,^39–43^ However, most of these studies were conducted in hospital settings. A meta-analysis showed a significant positive association between antibiotic resistance and antibiotic consumption at the community level with a pooled effect size (odds ratio) of 2.33 (z = 25.71, p 0.01, 95% confidence interval 2.19 to 2.49).^44^ Our study results showed no correlation between penicillin consumption and ampicillin resistance in commensal *E.coli*. However, while studying the changes in the ampicillin resistance rate, there was an observable rise in the resistance rate at the 6th and 7th-time points (Table 3), but no rise in penicillin consumption was noted during the same time period. But there was a notable rise in penicillin consumption at the 4th time point (Table 2). The rise in the resistance rate at the 6th and 7th-time points can be a lag effect of the increase in penicillin consumption at the 4th time point. However, we cannot determine whether the change was significant or not as the time period was short enough to show any interaction between antibiotic use and resistance.^45^ Our study also showed high resistance rates despite the reported total antibiotic consumption rate of only 45% in the study cohort, this could be the spillover effect, which has been shown to have consequences at the family and community level, where antibiotic use in one population selectively develops resistance that is passed to the other.^46^

#### Methodological Considerations

Our study has the following strengths: the study analyzed the association between the individual child-level consumption of antibiotics and resistance, at the community level in a defined cohort over a prospective longitudinal period. We collected the prospective data for antibiotic consumption and resistance and could analyse the relationship with real-life observations, over time. The reporting of antibiotic consumption was validated by twice-weekly follow up of the study cohort, which has enabled analysis of individual drug consumption to be correlated with antibiotic resistance. The information on antibiotic consumption was not self-reported by the caregivers. However, it was collected by actual pill count. During the HSB follow-ups, the antibiotic consumption data was recorded repeatedly from the same group of caregivers (caregivers of 125 children), which increases the reliability of the data and also helps control the factors that contribute to the variation in responses. Moreover, the recall period of 2 days lowered the likelihood of recall bias.

Our study has some limitations also as: the ATC/DDD approach was used in the study because it is the best available tool for comparing antibiotic use across different settings (communities, hospitals and national boundaries), as well as antibiotic use trends over time. However, there is only one DDD assigned to each generic compound, so this approach does not account for the fact that a patient’s age, diagnosis, and degree of sickness all have an effect on the appropriate dose. Furthermore, the DDD methodology does not provide pediatric doses. So, all DDDs were calculated and adjusted to per 100 patient-days and were also adjusted for the pediatric doses. Another limitation of our study is that for some of the antibiotics consumed we did not have ASTs, this was because AST was decided before the start of the study and adding some of the AST was not feasible.

## Conclusion

To conclude, we showed that there is no correlation between antibiotic consumption and the subsequent development of resistance in commensal *E.coli* at the rural community setting for the cephalosporin group of antibiotics. Further studies are needed to understand the factors causing the spillover effect like factors related to family, community and environment. We believe that effective antimicrobial stewardship strategies and antibiotic prescribing guidelines are needed at the local level, where our data on the association between antibiotic resistance rates and antibiotic consumption at the community level can be very useful. 

## Abbreviations

*E.coli*, Escherichia coli; WHO, World Health Organization; DDD, Defined daily doses; DSS, Demographic surveillance site; RDGMC, Ruxmaniben Deepchand Gardi Medical College; MP, Madhya Pradesh; CRL, Central research laboratory; PCR, Polymerase chain reaction; CLSI, Clinical and laboratory standards institute; AST, Antibiotic susceptibility test; HSB, Healthcare-seeking behaviour; ATC, Anatomical therapeutic chemical classification; DU, Drug use; SE, Standard error; ARTI, Acute respiratory tract infections.
